# MicroRNA-26a and -26b inhibit lens fibrosis and cataract by negatively regulating Jagged-1/Notch signaling pathway

**DOI:** 10.1038/cdd.2016.152

**Published:** 2017-06-16

**Authors:** Xiaoyun Chen, Wei Xiao, Weirong Chen, Xialin Liu, Mingxing Wu, Qu Bo, Yan Luo, Shaobi Ye, Yihai Cao, Yizhi Liu

**Affiliations:** 1 State Key Laboratory of Ophthalmology, Zhongshan Ophthalmic Center, Sun Yat-sen University, 54S Xianlie Road, Guangzhou 510060, People’s Republic of China; 2 Department of Microbiology, Tumor and Cell Biology, Karolinska Institute, 17177 Stockholm, Sweden

## Abstract

Fibrosis is a chronic process involving development and progression of multiple diseases in various organs and is responsible for almost half of all known deaths. Epithelial–mesenchymal transition (EMT) is the vital process in organ fibrosis. Lens is an elegant biological tool to investigate the fibrosis process because of its unique biological properties. Using gain- and loss-of-function assays, and different lens fibrosis models, here we demonstrated that microRNA (miR)-26a and miR-26b, members of the miR-26 family have key roles in EMT and fibrosis. They can significantly inhibit proliferation, migration, EMT of lens epithelial cells and lens fibrosis *in vitro* and *in vivo*. Interestingly, we revealed that the mechanisms of anti-EMT effects of miR-26a and -26b are via directly targeting Jagged-1 and suppressing Jagged-1/Notch signaling. Furthermore, we provided *in vitro* and *in vivo* evidence that Jagged-1/Notch signaling is activated in TGF*β*2-stimulated EMT, and blockade of Notch signaling can reverse lens epithelial cells (LECs) EMT and lens fibrosis. Given the general involvement of EMT in most fibrotic diseases, cancer metastasis and recurrence, miR-26 family and Notch pathway may have therapeutic uses in treating fibrotic diseases and cancers.

Fibrosis is a chronic multiple organ disease and is responsible for almost half of all known deaths. Some severe fibrosis cases even have a lower survival rate than cancer.^1^ Pathological epithelial–mesenchymal transition (EMT) is a critical element in organ fibrosis, such as pulmonary fibrosis, renal fibrosis, hepatic fibrosis and ocular fibrosis. In fibrotic tissues, myofibroblasts accumulate and secrete excessive collagen, thereby damaging organ function and leading to its failure.^2^ There is currently no specific therapy that targets fibroblast-associated pathology. It is essential to better understand the cellular and molecular mechanism of fibrotic event. Former researches have proposed that lens is an elegant biological tool to investigate the fibrosis processes because of its unique biological properties.^3^ Lens fibrotic disorders, such as anterior subcapsular cataract (ASC) and posterior capsule opacification (PCO), are common causes of visual impairment all over the world. Therefore, using the lens as a model for fibrosis not only has direct relevance to lens fibrotic disorders, but also serves as a valuable experimental tool to understand fibrosis essentially.

ASC and PCO are two types of cataract but share many cellular and molecular features.^4, 5^ ASC is a primary cataract, which is characterized by dense light-scattering fibrotic regions underneath the anterior capsule of the lens. It is mainly caused by ocular trauma, inflammation or irritation.^3^ PCO is known as a secondary cataract, a major long-term complication of cataract surgery, occurring in 20 to 40% of patients after surgery, particularly, almost 100% in children and infants.^6, 7^ The cellular mechanism of ASC and PCO is the proliferation, migration and EMT of lens epithelial cells (LECs), leading to the transition from epithelium to fibroblasts, and the production of extracellular matrix (ECM) proteins (collagens I, IV and fibronectin), which finally contributes to the formation of subcapsular plaques beneath the lens anterior or posterior capsule.^4, 8^ TGF*β*, especially TGF*β*2, the major isoform in the aqueous humor of the eye, is the most important factor in these processes.^9^ Hence, inhibition of LECs proliferation, migration and EMT may be a potential strategy to prevent ASC and PCO.

Aberrant Notch signaling has been found in a range of different cancers and fibrotic diseases.^10, 11, 12, 13^ The inactivation of Notch signaling is important in overcoming drug resistance and the reversal of EMT phenotype, which may improve the overall survival of cancer and fibrosis patients. In mammals, there are four cell surface transmembrane receptors (Notch-1–4) and five ligands (Jagged-1, Jagged-2, Delta-like 1, Delta-like 3 and Delta-like 4).^14^ On ligand binding, the Notch receptors are cleaved by *γ*-secretase, releasing Notch intracellular domain (NICD), which subsequently translocates into the nucleus and regulates downstream target genes,^15^ including Hes and Hey.^10^ Previous studies have identified that Jagged-1/Notch signaling is required for TGF*β*1-induced EMT in kidney and alveolar epithelial cells, using specific inhibitor of Notch signaling alleviates TGF*β*1-EMT, suggesting that pharmacological targeting of Notch pathway may be beneficial in the treatment of kidney fibrosis and pulmonary fibrosis.^16, 17, 18^ Despite its definite effects on EMT inhibition, the function of Notch signaling in LEC–EMT is currently unstated.

MicroRNAs (miRNAs, miRs) are small noncoding RNAs that negatively regulate gene expression at the posttranscriptional level. Through binding to complementary sequences in the 3′-untranslated regions (3′-UTR) of their target mRNAs, miRNAs can induce mRNA degradation or translation suppression.^19^ Accumulating evidence has demonstrated that miRNAs have important roles in EMT, such as miR-200 family,^20^ miR-29b,^21^ miR-34a^22^ and miR-491-5p.^23^ MiR-26 family comprises two mature miRNAs: miR-26a and miR-26b. They have been both reported to be tumor suppressors in hepatocellular carcinoma,^24^ breast cancer^25, 26^ and glioma development.^27^ MiR-26b can inhibit tumor cell growth and induce cell apoptosis by targeting PTGS2 and SLC7A11,^25, 26^ and suppress tumor cell migration and invasion by directly regulating EphA2.^27^ Regard to the roles of miR-26 family in fibrosis, Liang *et al.*^28^ has demonstrated the antifibrotic role of miR-26a by directly targeting Smad4, inhibits the nuclear translocation of p-Smad3, and further represses TGF*β*1-induced fibrogenesis in idiopathic pulmonary fibrosis. Wei *et al.*^29^ and Liang *et al.*^30^ also reported miR-26a can inhibit cardiac fibrosis and idiopathic pulmonary fibrosis by targeting collagen I, CTGF and HMGA2, respectively. The latest studies also demonstrated that miR-26b is significantly decreased in patient PCO tissues,^31^ and overexpression of it can inhibit LEC–EMT by targeting Smad4 and COX-2.^32^ However, the function of miR-26a in lens fibrosis is entirely unknown.

In the present study, we focused on miR-26a and -26b to further explore their functions in fibrosis using the lens as a model. We applied gain- and loss-of-function assays and different models to demonstrate miR-26a and miR-26b are EMT suppressors in lens fibrosis. Importantly, we revealed a novel mechanism underlying the functions of miR-26a and miR-26b by showing that they inhibit EMT via directly targeting Jagged-1 and suppressing Jagged-1/Notch signaling. Furthermore, using of Jagged-1 siRNA and Notch pathway specific inhibitor DAPT can also reverse LEC–EMT and ASC development. Our data suggest that miR-26 family and pharmacological targeting of Notch pathway may be of therapeutic value in the prevention and treatment of ASC, PCO and other organ fibrosis.

## Results

### Downregulation of miR-26a and -26b in TGF*β*2-induced EMT in LECs and the injury-induced ASC mouse model

**Figure 1:**
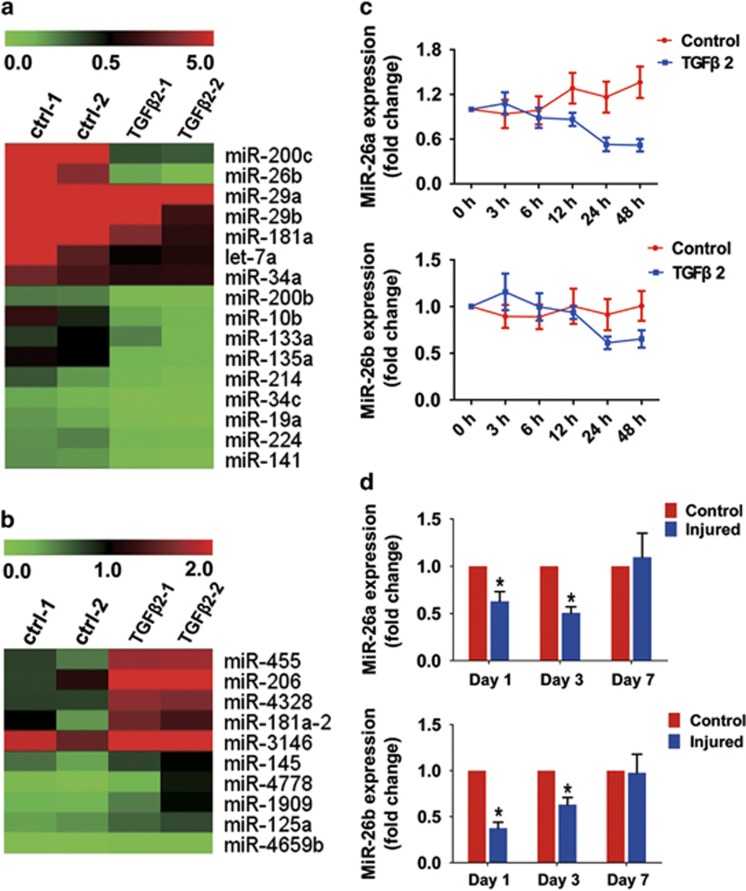
MiR-26a and miR-26b are downregulated in TGF*β*2-induced EMT in LECs and injury-induced ASC model in mice. (**a**) MiRNA array analysis shows the representative downregulated miRNAs in TGF*β*2-stimulated LECs for 48 h. The pseudocolor represents the intensity scale of the TGF*β*2 (5 ng/ml) treatment group versus the control group (*n*=3 per group). (**b**) MiRNA array analysis shows the representative upregulated miRNAs in TGF*β*2-stimulated LECs for 48 h. (**c**) Real-time PCR analysis of the expression of miR-26a and -26b in response to TGF*β*2 (5 ng/ml) in LECs at different time points. (**d**) Real-time PCR analysis of miR-26a and -26b in injury-induced ASC in mice at day 1, day 3 and day 7 after injury. *P<0.05

To determine which miRNA species may be involved in regulating LEC–EMT and the development of ASC, we first compared the miRNA expression profiles in TGF*β*2-induced LEC–EMT. Microarray analysis revealed that 101 miRNAs were elevated, and 223 were downregulated in TGF*β*2-induced LEC–EMT (Figures 1a and 1b). These miRNAs included miR-26b, which has been reported to be downregulated in human PCO tissues.^31^ Using real-time PCR analysis, we verified that miR-26b was reduced in response to TGF*β*2 exposure at 24 and 48 h by 35% and 39%, respectively. As miR-26a also belongs to the miR-26 family, we examined the miR-26a expression meanwhile. Real-time PCR analysis demonstrated that miR-26a was simultaneously downregulated in TGF*β*2-induced LEC–EMT (Figure 1c). In addition, we also validated the levels of miR-26a and -26b in injury-induced ASC model in mice. As expected, miR-26a and -26b were both rapidly decreased at day 1 and day 3 after injury, whereas at day 7, their expression returned to the baseline (Figure 1d). Collectively, these results indicate that miR-26a and -26b may have important roles in LEC–EMT and the development of ASC.

### MiR-26a and -26b inhibit LEC proliferation, migration and TGF*β*2-induced EMT

**Figure 2:**
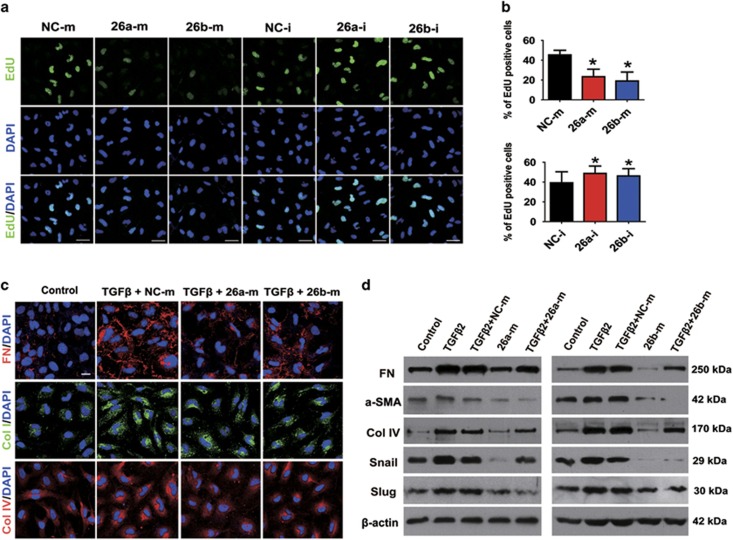
MiR-26a and -26b inhibit LECs proliferation and TGF*β*2-induced EMT *in vitro*. (**a**) EdU staining analysis of LECs proliferation after transfected with miRNA negative control mimic (NC-m), miR-26a mimic (26a-m), or miR-26b mimic (26b-m), miRNA negative control inhibitor (NC-i), miR-26a inhibitor (26a-i), or miR-26b inhibitor (26b-i) for 48 h, respectively. Scale bar, 40 *μ*m. (**b**) Quantification of EdU-positive cells (*n*=24 randomized fields per group); *P<0.05. (**c**) Immunofluorescent staining analysis of EMT markers FN, Col I and Col IV in LECs transfected with miRNA negative control mimic, miR-26a mimic or miR-26b mimic, and treated with TGF*β*2 (5 ng/ml) for 48 h. Scale bar, 20 *μ*m. (**d**) Western blot analysis of FN, *α*-SMA, Col IV, Snail and Slug protein levels in LECs transfected and treated as indicated in (**c**)

To understand the biological functions of miR-26a and -26b in LECs, we then transfected LEC cell line SRA01/04 with miR-26a and -26b mimic and inhibitor oligonucleotides, and examined LECs proliferation, migration and TGF*β*2-induced EMT by using gain- and loss-of-function experiments. Real-time PCR analysis confirmed that high and low levels of miR-26a and -26b expression were achieved in mimic- and inhibitor-transfected cells after 48 h compared with the negative control (NC)-transfected cells (Supplementary Figure S1a). Interestingly, EdU staining showed that the proliferations of LECs were markedly suppressed after transfection with miR-26a and -26b mimics, while increased in inhibitor-transfected cells (Figures 2a and b). Moreover, wound-healing assay demonstrated that overexpression of miR-26a and -26b both dramatically inhibited LECs migration, while downregulation of them induced LECs migration conversely (Supplementary Figures S1b and S1c). Subsequently, TGF*β*2 was applied to establish LEC–EMT model *in vitro*. As showed in Figure 2 and Supplementary Figure S2, the results of real-time RCR, western blot and immunofluorescence staining all showed that TGF*β*2 treatment induced EMT markers *α*-SMA, collagen type I (Col I), collagen type IV (Col IV) and fibronectin (FN), and EMT transcription factors Snail and Slug expression in LECs. Strikingly, overexpression of miR-26a and -26b both exhibited a significant downregulation of *α*-SMA, Col I, Col IV, FN, Snail and Slug induced by TGF*β*2. Conversely, knockdown of miR-26a and -26b upregulated *α*-SMA, FN, Snail and Slug without TGF*β*2 (Supplementary Figure S2b). These data suggest that miR-26a and -26b can significantly suppress LECs proliferation, migration and attenuate TGF*β*2-induced EMT *in vitro*.

### MiR-26a and -26b prevent injury-induced ASC in the mouse lens anterior capsular injury model

**Figure 3:**
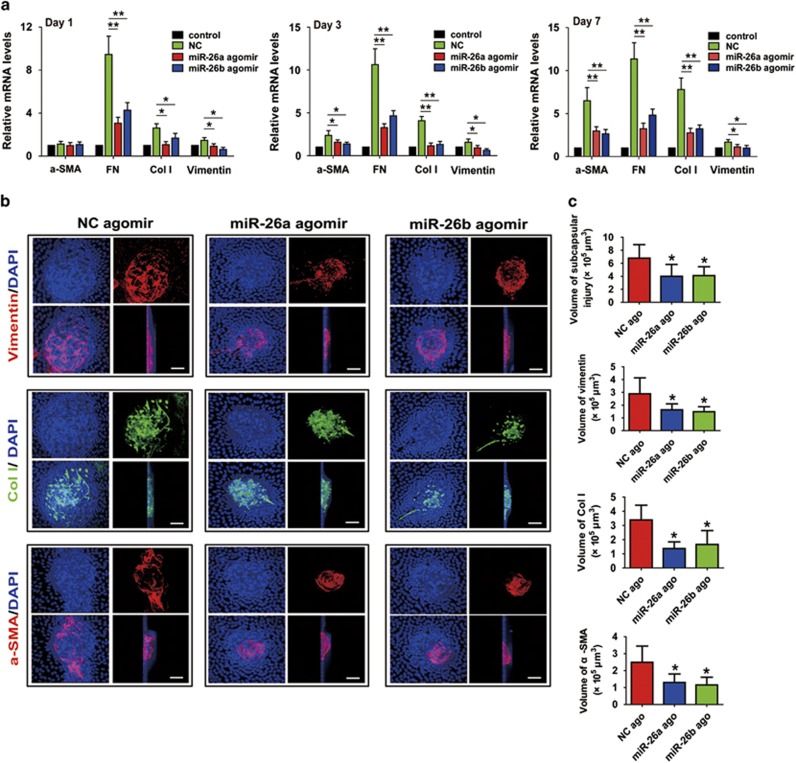
MiR-26a and -26b prevent injury-induced ASC *in vivo*. The anterior capsules of mouse lens were punctured with a needle and 1 *μ*l of 1 nM of miRNA negative control (NC) agomir, miR-26a agomir or miR-26b agomir were injected into the anterior chamber of the eye immediately after injury with a microsyringe. (**a**) One, 3 and 7 days later, lenses were harvested for real-time PCR analysis of *α*-SMA, FN, Col I and vimentin mRNA levels. *P<0.05; **P<0.01. (**b**) Representative confocal microscopy 3D images of lens capsule whole mounts show regions of subcapsular plaques, and EMT markers vimentin, Col I, and *α*-SMA staining after 7 days. Scale bar, 20 *μ*m. (**c**) Quantification of the subcapsular plaques volumes, and EMT markers vimentin, Col I and *α*-SMA distributions (*n*=6 lenses per group). *P<0.05

On the basis of the results *in vitro*, we next validated the anti-EMT roles of miR-26a and -26b *in vivo*. The mouse lens anterior capsular injury model is a well-established way for studying LECs proliferation, EMT, ECM components deposition and subcapsular plaque formation, as typically seen in human ASC and PCO. In this model, gain-of-function study was conducted using miR-26a and -26b agomirs, which were applied to stably induce endogenous expression of miRNAs *in vivo*. As expected, after miR-26a and -26b agomirs were injected into the anterior chamber of the mice, they could markedly induce the expression of miR-26a and 26b in the lens, and maintained for at least 7 days (Supplementary Figure S3). Strikingly, overexpression of miR-26a and -26b dramatically decreased EMT markers *α*-SMA, FN, Col I and vimentin expression induced by injury of lens anterior capsular for 1, 3 and 7 days (Figure 3a). Lens anterior capsule whole-mount staining also showed lenses developed remarkable multilayered LECs opacities around the capsular break and beneath the lens anterior capsule when the lenses were injured for 7 days, and strong staining of *α*-SMA, Col I and vimentin could be seen in the multilayered LECs opacities. However, in the miR-26a and -26b overexpression lens, the volumes of the subcapsular plaques and the expression of *α*-SMA, Col I and vimentin were obviously decreased compared with the NC agomir group (Figures 3b and c). Together, these findings further prove that miR-26a and -26b are negative regulators in LEC–EMT and ASC development *in vivo*.

### MiR-26a and -26b inhibit LEC–EMT via directly targeting Jagged-1 and suppressing Jagged-1/Notch signaling

**Figure 4:**
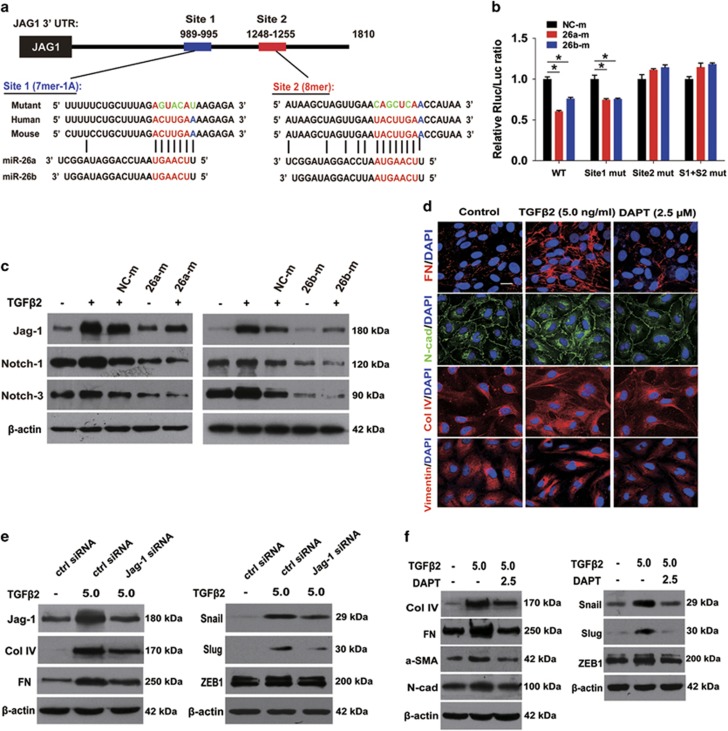
MiR-26a and -26b inhibit LEC–EMT via directly targeting Jagged-1/Notch signaling, and Jagged-1 siRNA and Notch pathway specific inhibitor DAPT reverse LEC–EMT *in vitro*. (**a**) Predicted miR-26a and -26b target two sites of potential-binding sequences in Jagged-1-3′UTR and mutants containing four mutated nucleotides (green) in two sites of Jagged-1-3′UTR, respectively. (**b**) Normalized luciferase activities of reporters containing wild-type or mutant 3′-UTR of Jagged-1 in 293 T cells co-transfected with miRNA negative control mimic (NC-m), miR-26a mimic (26a-m) or miR-26b mimic (26b-m). *P<0.05. (**c**) Western blot analysis of Jagged-1, Notch-1 and Notch-3 protein levels in LECs transfected with miRNA negative control mimic, miR-26a mimic or miR-26b mimic, and treated with TGF*β*2 (5 ng/ml) for 48 h. (**d**) Immunofluorescent staining analysis of EMT markers FN, N-cadherin, Col IV and vimentin in LECs transfected and treated as indicated in **c**. Scale bar, 40 *μ*m. (**e**) Western blot analysis of Jagged-1, Col IV, FN, Snail, Slug and ZEB1 protein levels in LECs transfected with control siRNA, or Jagged-1 siRNA, and treated with TGF*β*2 (5 ng/ml) for 48 h. (**f**) Western blot analysis of Col IV, FN, *α*-SMA, N-cadherin, Snail, Slug and ZEB1 protein levels in LECs exposure to TGF*β*2 with or without DAPT (2.5 *μ*M) for 48 h

To clarify the underlying mechanisms of the inhibitory effects of miR-26a and -26b on LEC–EMT, we used two miRNA target identification tools PicTar (http://pictar.mdc-berlin.de/) and TargetScan 4.2 (http://www.targetscan.org/vert_42/) to search for potential target genes of miR-26a and -26b. We found that Jagged-1 is a potential target of miR-26a and -26b (Figure 4a). In light of the critical role of the Jagged/Notch pathway in EMT during embryonic development, fibrotic diseases and cancer metastasis,^33, 34^ we then examined whether miR-26a and -26b inhibit LEC–EMT via regulating Jagged-1/Notch pathway. To confirm this, luciferase reporter carrying two miR-26a and -26b potential-binding sites or mutant-binding sites of Jagged-1 3′-UTR were constructed and co-transfected with miR-26a, -26b or NC mimic into the 293 T cells. Compared with the NC mimic group, transfection with miR-26a and -26b mimics reduced the luciferase activities significantly in the wild-type and site 1 mutant construct (Figure 4b). However, NC, miR-26a and -26b mimics did not affect the luciferase activities in the site 2 mutant and the site 1 plus site 2 mutant constructs (Figure 4b). These findings indicate that miR-26a and -26b directly interacts with the 3′-UTR of Jagged-1 via binding to site 2, but not site 1.

**Figure 5:**
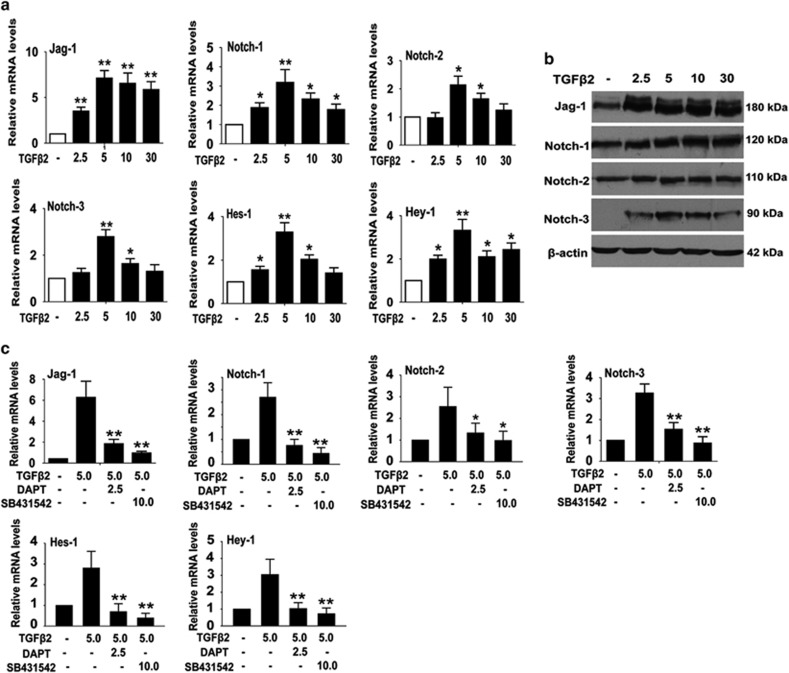
TGF*β*2 activates Jagged-1/Notch pathway in LECs EMT through canonical Smad2/3 signaling. (**a**) Real-time PCR analysis of Jagged-1, Notch-1, Notch-2, Notch-3, Hes-1 and Hey-1 mRNA levels in LECs treated with different concentrations of TGF*β*2 (2.5, 5, 10 and 30 ng/ml) for 48 h. *P<0.05; **P<0.01. (**b**) Western blot analysis of Jagged-1, Notch-1, Notch-2 and Notch-3 protein levels in LECs treated as indicated in (**a**). (**c**) Real-time PCR analysis of Jagged-1, Notch-1, Notch-2, Notch-3, Hes-1 and Hey-1 mRNA levels in LECs exposure to TGF*β*2 with DAPT (2.5 *μ*M) or SB431542 (10 *μ*M) for 48 h. *P<0.05; **P<0.01

Although Jagged-1/Notch pathway has been extensively studied in cancer and fibrotic diseases, the role of Notch signaling in LEC–EMT is largely unknown. Therefore, we next investigated whether Notch pathway is involved in TGF*β*2-stimulated EMT in LECs. As shown in Figure 5, TGF*β*2 could significantly induce the expression of Notch pathway ligand Jagged-1, receptors Notch-1, Notch-2 and Notch-3, and the downstream target genes Hes-1 and Hey-1. Jagged-1 was the most significantly upregulated gene in response to TGF*β*2 treatment. However, TGF*β*2-induced activation of Notch signaling was dramatically inhibited by SB431542, a specific inhibitor for TGF*β*/Smad2/3 signaling. These results suggest that TGF*β*2 activates Jagged-1/Notch pathway via the canonical TGF*β*/Smad2/3 signaling. Next, we confirmed whether miR-26a and -26b inhibit LEC–EMT via directly regulating Jagged-1/Notch signaling. The results from real-time RCR and western blot showed that the mRNA and protein levels of Jagged-1 were markedly decreased in miR-26a and -26b mimics-transfected LECs, but slightly increased in cells transfected with miR-26a and -26b inhibitors (Supplementary Figure S4 and Figure 4c). This implies that miR-26a and -26b regulate the expression of Jagged-1 at the transcriptional and the posttranscriptional levels. Besides, Notch-1 and Notch-3 were also reduced in miR-26a and -26b mimic-transfected cells (Figure 4c), while increased in inhibitor-transfected cells (Supplementary Figure S4d). Furthermore, real-time PCR results revealed that overexpression of miR-26a and -26b in injury-induced ASC model also suppressed Jag-1, Notch-1, Notch-2 and Notch-3 expression *in vivo* (Supplementary Figure S5). Taken together, these results reveal that the activation of Jagged-1/Notch pathway is involved in LEC–EMT, and the mechanism of the inhibitory effects of miR-26a and -26b on LEC–EMT is via directly targeting Jagged-1 and suppressing Jagged-1/Notch signaling.

### Jagged-1 siRNA and blockade of Notch signaling reverse TGF*β*2-induced EMT in LECs

To further explore the role of Jagged-1/Notch signaling in LEC–EMT, we study the impacts of blockade of Notch pathway via knockdown of Jagged-1 using siRNA and DAPT (a *γ*-secretase inhibitor) in TGF*β*2-induced LEC–EMT. As illustrated in Figure 4 and Supplementary Figure S6, inhibition of Notch signaling by Jagged-1 siRNA and DAPT both abrogated the upregulation of EMT markers FN, Col IV, N-cadherin, *α*-SMA and EMT key transcription factors Snail, Slug and ZEB1 expression. Furthermore, wound-healing and EdU-staining assays demonstrated that DAPT dramatically inhibited LECs migration, whereas had no effect on cell proliferation (Supplementary Figure S7). These data clearly demonstrate that downregulation of Jagged-1 and blockade of Notch pathway can reverse TGF*β*2-induced EMT phenotype in LECs.

### Blockade of Notch pathway abrogates TGF*β*2-induced ASC *in vitro* and injury-induced ASC *in vivo*

**Figure 6:**
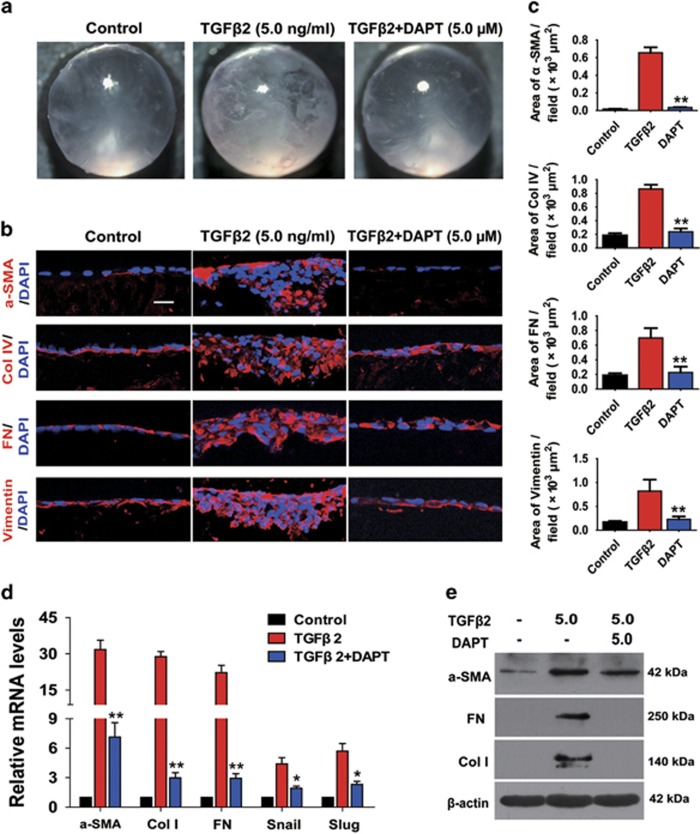
Blockade of Notch pathway with DAPT prevents TGF*β*2-induced ASC *in vitro*. Rat lenses were cultured in the absence or presence of TGF*β*2 with DAPT (5.0 *μ*M) or DMSO for 7 days. (**a**) Representative dissecting microscope images of the morphological changes of the lenses cultured for 7 days (*n*=12 lenses per group). (**b**) Representative confocal microscopy images of frozen sections staining for *α*-SMA, Col IV, FN and vimentin (*n*=6 lenses per group). Scale bar, 20 *μ*m. (**c**) Quantification of the area of *α*-SMA, Col IV, FN and vimentin per field (*n*=24 randomized fields per group). **P<0.01. (**d**) Seven days later, the lenses were harvested for real-time PCR analysis of *α*-SMA, Col I, FN, Snail and Slug mRNA levels (*n*=6 lenses per group). *P<0.05; **P<0.01. (**e**) Western blot analysis of *α*-SMA, FN and Col I protein levels in lenses

Previous studies have identified that TGF*β* can induce the whole lens cultured *in vitro* to form opacities that contain morphologic and pathological markers for ASC.^35^ To further investigate whether blockade of Notch signaling by DAPT reverses TGF*β*2-induced EMT in the lens in a more complicated system, we utilized the whole lens culture semi-*in vivo* model. When the lenses from 20- to 22-day-old rats were cultured with 5 ng/ml of TGF*β*2 for 7 days, the lenses developed obvious clumpy opacities beneath the lens capsule in each lens, whereas DAPT abrogated TGF*β*2-induced ASC and the lenses remained transparent as cultured without TGF*β*2 (Figure 6a). As shown in Figures 6b and c, the morphology of the frozen sections showed that the aberrant cells were co-localized with the subcapsular clumpy opacities. The clumps contained accumulations of FN and Col IV, and increases of *α*-SMA and vimentin expression. In contrast, the lenses cultured with DAPT retained normal lens morphology, and did not have obvious accumulations of FN, Col IV, *α*-SMA and vimentin. In addition, we also demonstrated that DAPT dramatically reduced the upregulation of *α*-SMA, Col I, FN, Snail and Slug at mRNA and the protein levels in rat lenses induced by TGF*β*2 for 7 days (Figures 6d and e).

**Figure 7:**
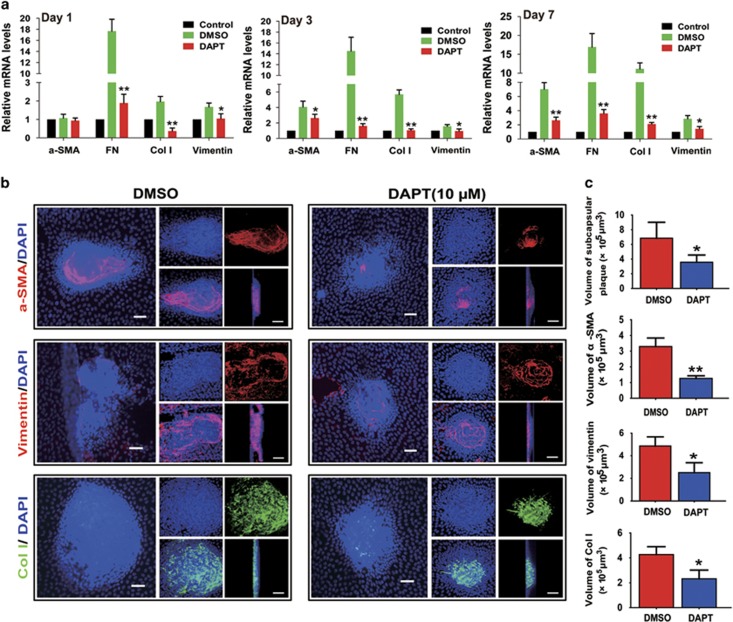
Blockade of Notch pathway with DAPT inhibits injury-induce ASC *in vivo*. The anterior capsules of mouse lens were punctured with a needle and 1 *μ*l of 80 *μ*M of DAPT were injected into the anterior chamber of the eye immediately after injury. (**a**) One, 3 and 7 days later, the lenses were harvested for real-time PCR analysis of *α*-SMA, FN, Col I and vimentin mRNA levels. *P<0.05; **P<0.01. (**b**) Representative fluorescence microscope (left column) and confocal microscopy 3D (right column) images of lens capsule whole mounts show regions of subcapsular plaques, and EMT markers *α*-SMA, vimentin and Col I staining at 7 day after injury. Scale bar, 40 *μ*m in left column; 20 *μ*m in right column. (**c**) Quantification of the subcapsular plaques volumes, and EMT markers *α*-SMA, vimentin and Col I distributions (*n*=6 lenses per group). *P<0.05; **P<0.01

Finally, we used injury-induced mouse ASC model to further verify the inhibitory effects of DAPT on ASC *in vivo*. First, we found Notch pathway ligands Jagged-1 and Jagged-2, receptors Notch-1, Notch-2 and Notch-3, and the downstream target genes Hes-1 and Hey-1 were obviously upregulated from day 1 to day 3 after injury (Supplementary Figure S8), and returned to the base lines at day 5 (data not shown). These results show that Notch pathway is actually required for injury-induced EMT of lens epithelium. Next, the results from real-time PCR displayed DAPT treatment notably decreased EMT markers *α*-SMA, FN, Col I and vimentin expression when the lenses were injured for 1, 3 and 7 days (Figure 7a). Immunofluorescent staining of the lens anterior capsule whole-mounts also showed the volumes of the subcapsular plaques and the expression of *α*-SMA, Col I and vimentin in the subcapsular plaques were obviously decreased in DAPT treated lenses (Figure 7b). Taken together, these findings indicate that Notch signaling has a vital role in the development of ASC, inhibition of Notch signaling can suppress ASC formation *in vitro* and *in vivo*.

## Discussion

**Figure 8:**
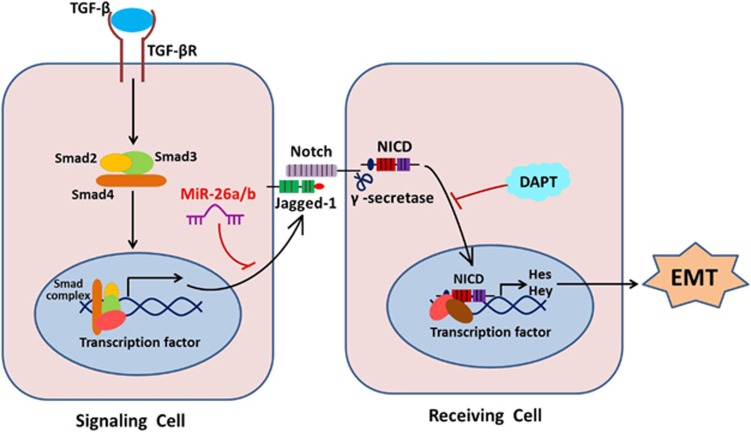
Schematic illustration of the functions of miR-26a/b-Jagged/Notch axis in modulation of EMT and fibrosis. During the EMT process, TGF*β*2 can activate Jagged-1/Notch pathway via the canonical Smad2/3 signaling. Upon ligand binding, the Notch receptors are cleaved by *γ*-secretase, releasing NICD, which subsequently translocates into the nucleus and regulates downstream target genes expression, leading to EMT and fibrosis. Nevertheless, miR-26a and -26b can directly target Jagged-1 and suppresses Jagged-1/Notch signaling, thus inhibiting EMT and fibrosis. Meanwhile, knockdown of Jagged-1 and using Notch pathway specific inhibitor DAPT can also reverse EMT and fibrosis

Fibrosis occurs in almost all tissues and organs, which under pathological conditions often impairs organ functions and in some cases significant morbidity and mortality. MiR-26 family has been verified that they have antifibrotic effects in idiopathic pulmonary fibrosis and cardiac fibrosis,^28, 29, 30^ however, its biological effects on LEC–EMT progression and lens fibrosis remain unclear. Our results from cultured cells *in vitro* and injury-induced ASC model *in vivo* revealed that Jagged-1/Notch pathway have a crucial role in LEC–EMT and lens fibrosis. MiR-26a and -26b are EMT and fibrosis suppressors. They markedly inhibit LECs proliferation, migration and EMT, and the development of injury-induced ASC in mice. Notably, we revealed a previously unidentified mechanism that miR-26a and -26b inhibit EMT via directly targeting Jagged-1 and negatively regulating Jagged-1/Notch signaling. Furthermore, blockade of Notch pathway can also reverse LEC–EMT and lens fibrosis (Figure 8).

MiR-26 family has been reported to be fibrosis suppressors in idiopathic pulmonary fibrosis and cardiac fibrosis,^28, 29, 30^ however, its role in lens fibrosis is completely unknown. In this study, we found that the expression of miR-26a and -26b were decreased in TGF*β*2-induced LEC–EMT. Analysis of the promoter sequence showed there is a potential-binding site for Smad3 in the region upstream of the miR-26a promoter. Previous study has already verified the effect of TGF*β* on miR-26a is in fact mediated by Smad3.^28^ Activation of Smad3 can significantly repress miR-26a expression, meanwhile, knockdown of Smad3 increases miR-26a expression and reverses the downregulation of miR-26a induced by TGF*β*. These results indicated that Smad3 negatively regulates transcription of miR-26a.^28^ Moreover, the complex of Smad2/Smad3/Smad4 also possibly bind to the promotor of miR-26b. Smad4 has been reported to be the target gene of miR-26b,^32^ so miR-26b downregulation results in upregulation of Smad4, which increases nuclear translocation of p-Smad3 and may further suppresses miR-26b. Therefore, we think miR-26a and -26b downregulations are probably the direct effects of TGF*β*. Futhermore, miR-26a and miR-26b were also decreased in injury-induced ASC in mice, which suggesting they may be associated to lens fibrosis.

To gain in-depth understanding of biological functions of miR-26a and -26b in lens fibrosis, we examined their effects on LECs proliferation, migration and EMT using gain- and loss-of-function experiments. Our EdU-staining and wound-healing assays both demonstrated that miR-26a and -26b markedly suppress LECs proliferation and migration. In addition, they exhibit significant inhibition of TGF*β*2-induced LEC–EMT *in vitro*. These are consistent to the previous study.^32^ With regard to miR-26a, we reported, for the first time, that it suppresses LECs proliferation, migration and EMT as well as miR-26b. Importantly, we further investigated the anti-EMT effects of miR-26a and -26b *in vivo* using miRNA agomirs. In injury-induced ASC model, miR-26a and -26b agomirs were injected into the anterior chambers of the mouse eyes for the first time. Excitingly, they can successfully induce the expression of miR-26a and -26b in the lens, and maintain for 7 days. Using this model, we found overexpression of miR-26a and -26b dramatically suppressed injury-induced LEC–EMT, ECM components deposition and the development of ASC *in vivo*. Therefore, miR-26a and -26b both can inhibit LECs proliferation, migration and EMT, and further inhibit injury-induced ASC *in vitro* and *in vivo*.

Several target genes of miR-26a and -26b have been identified for fibrosis thus far, including collagen I, CTGF, Smad4 and HMGA2.^28, 29, 30^ In the current study, we uncovered a novel target gene Jagged-1 for miR-26a and -26b. We confirmed that they can directly interact with the 3′-UTR of Jagged-1, thus negatively regulate Jagged-1 expression. Interestingly, the Notch receptors Notch-1 and Notch-3 were also reduced in the miR-26a and -26b mimic-transfected cells. This implies that miR-26a and -26b inhibit LEC–EMT via directly targeting Jagged-1 and suppressing Jagged-1/Notch signaling. Notch signaling has been reported to have a critical role in EMT during embryonic development, cancer metastasis and various fibrotic diseases.^33^ However, its functions in LEC–EMT remains poorly understood. Our results demonstrated that Notch pathway is activated by TGF*β*2 in LECs through the canonical TGF*β*/Smad signaling, and Jagged-1 is the most upregulated gene by TGF*β*2 (Figure 8). Subsequently, we found inhibition of Notch signaling entirely reversed TGF*β*2-induced LEC–EMT *in vitro* and TGF*β*2-induced ASC formation in the whole lens culture semi-*in vivo* model. We also further confirmed Notch pathway was actually activated in injury-induced EMT of lens epithelium, and DAPT treatment obviously inhibited injury-induced LEC–EMT and the development of ASC *in vivo*. Taken together, our data indicate that the activation of Notch signaling has a vital role in LEC–EMT and ASC formation, inactivation of Notch signaling can be useful for the abrogation of LEC–EMT and ASC. It is noteworthy that miR-26a and -26b can both dramatically inhibit LECs proliferation, but blockade of Notch pathway with DAPT has no effect on the proliferation of LECs. This implies that miR-26a and -26b may inhibit the proliferation of LECs via targeting other genes. Such a possibility warrants future studies to explore the mechanism of effect of miR-26 family on cell proliferation.

In summary, using different gain- and loss-of-function assays and multiple model systems, our results provide, for the first time, evidence that miR-26 family and Jagged-1/Notch pathway have important roles in lens fibrosis. MiR-26a and -26b inhibit LECs proliferation, migration, EMT and ASC formation *in vitro* and *in vivo* via directly targeting Jagged-1 and negatively regulating Jagged-1/Notch signaling. Our data suggest that miR-26 family and blockade of Notch pathway may be promising strategies in the prevention and treatment of organ fibrosis. These insights into the regulatory relationship between miR-26 family and Notch signaling pathway are not only helpful in understanding the pathogenesis of fibrosis diseases, but also beneficial for studying cancer metastasis, drug resistance and recurrence.

## Materials and Methods

### Cell culture and treatment

The human LEC line SRA01/04 was kindly provided by Professor Fu Shang at the Laboratory for Nutrition and Vision Research, Tufts University (Boston, MA, USA), and cultured in Dulbecco’s modified Eagle’s medium (DMEM, Gibco, Life Technologies, NY, USA) containing 10% fetal bovine serum (FBS, Gibco, Life Technologies). For TGF*β*2 treatment, the cells were seeded in six-well plates and incubated with 5 ng/ml of TGF*β*2 (Cell Signaling, Danvers, MA, USA) for 48 h. Pharmacological inhibitors DAPT (a specific inhibitor of Notch receptor cleavage, Sigma-Aldrich, Louis, MO, USA) and SB431542 (a specific inhibitor of TGF*β*/Smad2/3 signaling, Sigma-Aldrich) were added 60 min before treatment with TGF*β*2.

### Microarray analysis

The preparation of RNA samples and microarray analysis were performed commercially by RiboBio Co. Ltd (Guangzhou, China). In brief, total RNA of LECs treatment with or without TGF*β*2 was isolated using TRIzol (Invitrogen, Carlsbad, CA, USA) and miRNeasy mini kit (QIAGEN, Hilden, Germany) according to the manufacturer’s instructions, which efficiently recovers all RNA species, including miRNAs. RNA quality and quantity were measured by nanodrop spectrophotometer (ND-1000, Nanodrop Technologies, Wilmington, DE, USA), and RNA integrity was determined by gel electrophoresis. The isolated miRNAs were then labeled with Hy3/Hy5 using the miRCURY Array Power Labeling kit (Exiqon, Vedbaek, Denmark) and hybridized on a miRCURY LNA miRNA Array (v.18.0, Exiqon) according to array manual. Following hybridization, the slides were achieved, washed several times using wash buffer kit (Exiqon) and finally dried by centrifugation for 5 min at 400 r.p.m. Then the slides were scanned using the Axon GenePix 4000B microarray scanner (Axon Instruments, Foster City, CA, USA). The scanned images were then imported into GenePix Pro 6.0 software (Axon Instruments) for grid alignment and data extraction. Bioinformatics analysis and visualization of microarray data were performed with MEV software (v4.6, TIGR). The microarray assays were repeated three times each group.

### Model of injury-induced ASC in mouse

All animal experiments were approved by the Animal Use and Care Committee of Zhongshan Ophthalmic Center at the Sun Yat-Sen University, Guangzhou, People’s Republic of China. The model of injury-induced ASC in mouse eyes were performed as described previously.^36, 37^ Before making the injury, the mice were anesthetized generally with intraperitoneal injection of pentobarbital sodium (70 mg/kg) and topically with dicaine eye drop. After pupillary dilation with compound tropicamide eye drop, a small incision was made in the central anterior capsule with the blade part of a 26-gauge hypodermic needle through the cornea in the right eye of the mouse. The depth of injury was approximately 300 *μ*m or one-fourth of the length of the blade part of the needle. To forced expression of miR-26a and -26b in this model, the mice were randomly divided into three groups to receive 1 *μ*l of 1 nM of miR-26a, miR-26b and NC agomirs (RiboBio; *n*=30 mice per group). The agomirs were injected into the anterior chambers of the injured eyes immediately after injury with a microsyringe (30-gauge, Hamilton). For DAPT treatment, the mice were randomly divided into two groups to receive 1 *μ*l of 80 *μ*M of DAPT and equivalent amount of DMSO as a control. The animals were allowed to heal for 1, 3 and 7 days. At the end of the treatment period, the mice were killed and the lenses were harvested for real-time PCR and whole-mount staining.

### Forced expression and knockdown of miR-26a and -26b *in vitro*

To examine the functions of miR-26a and -26b *in vitro*, gain- and loss-of-function experiments were performed. MiRNA mimics and inhibitors were applied to enhance or knockdown the endogenous expression of miR-26a and -26b according to the manufacturer’s protocols. For overexpression of miR-26a and -26b, when LECs grew to 60–70% confluency, the mixture of 100 nM of negative control (NC), miR-26a or -26b mimics (RiboBio) with 5 *μ*l of Lipofectamine 2000 (Invitrogen) was added into the cells in Opti-MEM medium. Conversely to knockdown of miR-26a and -26b, the cells were transfected with 200 nM of NC, miR-26a or -26b inhibitors (RiboBio) with 10 *μ*l of Lipofectamine 2000. After 4–6 h incubation, the medium was replaced and LECs exposed to 5 ng/ml of TGF*β*2 for a further 48 h.

### Wound-healing assay

After miR-26a and -26b mimics or inhibitors transfection, streaks were created with a 100 *μ*l yellow micropipette tip. For DAPT treatment, LECs were further incubated with or without DAPT (2.5 *μ*M) for 48 h. Progression of migration was monitored and photographed with an inverted phase contrast microscope after incubation for 48 h. For quantitative assessment, the remaining area of the wound in each image was determined by Image-Pro Plus software 5.1 (Media Cybernetics, Inc. Silver Spring, MD, USA).

### EdU-staining assay

LECs proliferation was determined using Cell-Light EdU Apollo 488 *In Vitro* Kit (RiboBio) according to the manufacturer’s protocols. In brief, SRA01/04 cells were seeded in six-well plates and transfected with miR-26a and -26b mimics or inhibitors for 48 h. The culture medium was replaced with fresh medium containing 50 *μ*M EdU for 2 h. After that, the cells were fixed with acetone for 10 min and washed with PBS, and then they were sequentially incubated with Apollo reaction buffer containing FITC-fluorescein for 30 min. Finally, the cells were incubated with DAPI, mounted and examined by a confocal microscopy (LSM510, Carl Zeiss, Overkochen, Germany).

### Real-time PCR analysis for gene expression

Total RNA from cultured cells and lenses was extracted using the Trizol reagent according to the manufacturer’s instruction. Then genomic DNA was removed using DNase I. For miRNAs, their cDNAs were synthesized using Mir-X miRNA First-Strand Synthesis kit (Clontech Laboratories, Mountain View, CA, USA), and the expression levels of miR-26a and -26b were quantified by real-time PCR using SYBR Premix Ex Taq II kit (Takara, Siga, Japan). Other gene cDNAs were synthesized using PrimeScript RT Master Mix kit (Takara) and their mRNAs expression were quantitatively analyzed by SYBR PrimeScript RT-PCR kit (Takara). Real-time PCR reactions were performed using an ABI Prism 7000 sequence detection system (Applied Biosystems, Foster City, CA, USA). RNU6B and glyceraldehyde 3-phosphate dehydrogenase (GAPDH) were used as internal controls. Human, rat and mouse primers used in this study were listed in Supplementary Table S1.

### Western blot analysis for protein expression

The cells were washed twice with PBS and then lysed in 100 *μ*l of lysis buffer with protease inhibitor cocktail for total protein extraction. The protein samples mixed with 5 × SDS sample buffer were subjected to SDS-PAGE, and then electroblotted onto PVDF membranes. The membranes were blocked in 5% non-fat milk and incubated with different primary antibodies at 4 °C overnight. After being washed with PBS containing 0.1% Tween20 (PBST), the membranes were incubated with horseradish peroxidase (HRP)-conjugated secondary antibodies for 1 h at room temperature (RT) and washed three times with PBST. The bands on the membranes were visualized using the chemiluminescence detection reagents.

The primary antibodies against Jagged-1, Notch-1, Notch-2, Notch-3, p-Smad2, p-Smad3, Snail, Slug, ZEB1, horse anti-mouse and goat anti-rabbit HRP-conjugated secondary antibodies were obtained from Cell Signaling Technology. Antibodies against *β*-actin, fibronetin (FN), collagen type I (Col I), collagen type IV (Col IV), N-cadherin (N-cad), *α*-SMA and vimentin were obtained from Abcam (Cambridge, UK).

### Immunofluorescence staining for cryosections and cultured cells

Lens cryosections or slides of cultured cells were fixed with acetone for 15 min at RT, permeated with 0.5% Triton X-100 for 10 min and blocked with 1% bovine serum albumin (BSA) for 1 h. Afterwards, the sections or cell slides were incubated with different primary antibodies at 4 °C for overnight in a humidity chamber. Next day, secondary antibodies Alexa Fluor 488-conjugated goat anti-rabbit or Alexa Fluor 555-conjugated donkey anti-mouse antibodies (Cell Signaling Technology) were incubated for 1 h at RT. After washing with PBS, the lens cryosections or cell slides were incubated with DAPI for nuclear staining and mounted with anti-fade mounting medium. The slides were observed using a laser scanning confocal microscopy (LSCM; LSM510, Carl Zeiss).

### Lens anterior capsule whole-mount staining

Lens anterior capsule whole-mount staining was performed according to our previously described method.^37^ Briefly, the injured mice were killed and their eyes were enucleated. And then the lens were isolated under a dissecting microscope and fixed in 100% methanol for 1 h at RT. After that, lenses anterior capsules were separated, and blocked and permeated with 1% Triton X-100 in 1% BSA for 1 h at RT. Primary antibodies diluted in PBS were added to immerse the capsules and incubated for 12–24 h at 4 °C. On the following day, after being washed with PBST, the capsules were incubated with appropriate secondary antibodies for 1 h at RT. After being stained with DAPI, the whole anterior capsules were mounted in anti-fade mounting medium on a microscope slide and examined using a fluorescence microscope (Carl Zeiss) and LSCM within a few days. For 3D images analysis, consecutive images of lens anterior capsule whole mount containing the whole anterior subcapsular plaque were acquired from LSCM, and then reconstructed using the Zeiss LSCM Image Browser software.

### Quantitative analysis of the subcapsular plaques and EMT marker distribution in injury-induced ASC model

Quantitative analysis of the sizes of the subcapsular plaques and EMT markers distribution was performed using LSCM Image Browser software as our method previously described.^37^ The shape of the subcapsular plaque in this model is similar to the trustum of a pyramid, so the volume of the subcapsular plaque in every two images can be calculated according to the formula of the volume of pyramid *V*_1_=1/3 × *H* × [*S*_up_+*S*_down_ +√(*S*_up_ × *S*_down_)], and the total volume of the subcapsular plaque in one sample equal to the sum total of every two images (*V*_total_=*V*_1_+*V*_2_+……+*V*_n_).^37^ A minimum of six capsules were analyzed for each group.

### Jagged-1 3′-UTR luciferase reporter assay

The 3′-UTR region of human Jagged-1 was amplified by PCR from genomic DNA and cloned downstream to the firefly luciferase coding region in the pMIR-RB-REPORT. The 293 T cells were seeded in 96-well plates 24 h before transfection. The following day, to access the effects of miR-26a and -26b on reporter activities, 100 ng/ml of reporter plasmid and 50 nM of miR-26a, -26b or NC mimics were co-transfected into 293 T cells using lipofectamine 2000. Luciferase activities were measured 48 h after transfection using the Dual-Glo Luciferase Assay System (Promega, Madison, WI, USA). All experiments were performed in triplicates.

### Jagged-1 siRNA knockdown in cultured cells *in vitro*

Jagged-1 siRNA was transfected into LECs with transfection reagent (Santa Cruz Biotechnology, Santa Cruz, CA, USA) according to the manufacturer’s instructions. In short, 60 nM of control siRNA or Jagged-1 siRNA was mixed with 6 *μ*l of transfection reagent in transfection medium and incubated for 30 min at RT. The mixture then was added into each well of the six-well plates containing cells in transfection medium, and the cells were incubated for 5–7 h. The transfection medium was subsequently replaced and the cells exposed to 5 ng/ml of TGF*β*2 for a further 48 h. For siRNA knockdown efficiency, the mRNA and protein levels of Jagged-1 were determined by real-time PCR and western blot, respectively.

### Model of TGF*β*2-induced ASC in whole lens culture

Lenses of 20–22 days old Wistar rats were cultured as described previously.^35, 38^ Shortly, whole lenses were carefully isolated and maintained in 4 ml serum-free M199 medium (two lenses per well) containing 0.1% BSA, 0.1 mg/ml l-glutamine, 50 IU/ml penicillin and 50 mg/ml streptomycin. Next day, cloudy lenses caused by technique were removed and TGF*β*2 was added to the culture medium at a final concentration of 5 ng/ml, using 0.1% BSA as a control. The culture medium was renewed every second day throughout the culture period. The lenses were cultured for up to 7 days and photographed using a dissecting microscope (Carl Zeiss). After that, the lenses were harvested for real-time PCR, western blot and cryosection staining.

### Statistical analysis

The results presented in the figures are representative of three or more different repetitions. All data are presented as mean±standard error of the mean (S.E.M.). The data were analyzed by using two-tailed Student's *t*-test and one-way analysis of variance (ANOVA) statistical analysis. A value of *P*<0.05 was considered statistically significant, *P*<0.01 was highly significant.
