# MicroRNA-26a and -26b inhibit lens fibrosis and cataract by negatively regulating Jagged-1/Notch signaling pathway

**DOI:** 10.1038/cdd.2017.147

**Published:** 2017-09-22

**Authors:** Xiaoyun Chen, Wei Xiao, Weirong Chen, Xialin Liu, Mingxing Wu, Qu Bo, Yan Luo, Shaobi Ye, Yihai Cao, Yizhi Liu

**Correction to:** *Cell Death and Differentiation* (2017) **24,** 1431–1442; doi:10.1038/cdd.2016.152; published online 16 June 2017

This article was originally published under a standard license, but has now been made available under a CC BY 4.0 license. The PDF and HTML versions of the paper have been modified accordingly.

