# Supplementary material for: MicroRNA-26a and -26b inhibit lens fibrosis and cataract by negatively regulating Jagged-1/Notch signaling pathway
Source: Cell Death Differ. 2017 Jun 16;24(8):1431–42. doi: 10.1038/cdd.2016.152 (PMC5520447; doi:10.1038/cdd.2016.152)
Supplement: Supplementary Information [file cdd2016152x1.docx]

**Supplemental information summary**

1. Supplemental information summary and legends of supplemental figures (Supplementary information. doc)
2. Supplemental figures (Figure S1-S8)

**Titles and legends to supplementary figures**

**Figure S1 MiR-26a and -26b** **inhibit LECs migration *in vitro***. (**a**) Real-time PCR analysis of the levels of miR-26a and -26b in LECs transfected with miRNA negative control mimic (NC-m), miR-26a mimic (26a-m), or miR-26b mimic (26b-m), miRNA negative control inhibitor (NC-i), miR-26a inhibitor (26a-i), or miR-26b inhibitor (26b-i) for 48 h, respectively. Mean values and SDs from three indepentdent experiments are shown. ** *P*<0.01 *vs* the NC-m or NC-i group. (**b**) Wound healing analysis of LECs migration after transfected as indicated in ***a***. Scale bar: 100 μm. (**c**) Quantification of the area of the remaining wound per field (n=18 randomized fields per group). Mean values and SDs from three indepentdent experiments are shown. * *P*<0.05 *vs* the NC-m or NC-i group.

**Figure S2 MiR-26a and -26b inhibit TGFβ2-induced LECs EMT *in vitro*.** (**a**) Real-time PCR analysis of the mRNA levels of EMT markers FN, Col I, Col IV, Snail and Slug in LECs transfected with miRNA negative control mimic, miR-26a mimic, or miR-26b mimic, and treated with TGFβ2 (5 ng/ml) for 48 h. * *P*<0.05. (**b**) Western blot analysis of α-SMA, FN, Snail, and Slug protein levels in LECs transfected with miRNA negative control inhibitor, miR-26a inhibitor, or miR-26b inhibitor for 48 h.

**Figure S3** **MiR-26a and -26b agomirs stably induce endogenous expression of miR-26a and -26b in injury-induced ASC model *in vivo.*** The anterior capsules of mouse lenses were punctured with a needle, and 1μl of 1nM of miRNA negative control (NC) agomir, miR-26a agomir, or miR-26b agomir were injected into the anterior chambers of the eyes with a microsyringe immediately after injury. One, three and seven days later, lenses were harvested for real-time PCR analysis of the levels of miR-26a and -26b. ** *P*<0.01.

**Figure S4** **MiR-26a and -26b negatively regulate Jagged-1/Notch signaling expression *in vitro*.** (**a**) Real-time PCR analysis of the mRNA level of Jagged-1 in LECs transfected with miRNA negative control mimic, miR-26a mimic, or miR-26b mimic, and treated with TGFβ2 (5 ng/ml) for 48 h. * *P*<0.05; ** *P*<0.01. (**b**) Real-time PCR analysis of Jagged-1 mRNA level in LECs transfected with miRNA negative control inhibitor, miR-26a inhibitor, or miR-26b inhibitor for 48 h. * *P*<0.05. (**c**) Western blot analysis of Jagged-1 protein level in LECs transfected as indicated in ***b***. (**d**) Western blot analysis of Notch-1and Notch-3 protein levels in LECs transfected as indicated in ***b***.

**Figure S5** **MiR-26a and -26b negatively regulate Jagged-1/Notch signaling in injury-induced ASC model *in vivo*.** The anterior capsules of mouse lens were punctured with a needle and 1μl of 1nM of miRNA negative control (NC) agomir, miR-26a agomir, or miR-26b agomir were injected into the anterior chambers of the eyes immediately after injury with a microsyringe. Three days later, lenses were harvested for real-time PCR analysis of Jag-1, Notch-1, Notch-2, and Notch-3 mRNA levels. * *P*<0.05.

**Figure S6 Jagged-1 siRNA and Notch pathway specific inhibitor DAPT reverse LECs EMT *in vitro***. (**a**) Real-time PCR analysis of Jagged-1, Col IV, FN, and N-caherin mRNA levels in LECs transfected with control siRNA, or Jagged-1 siRNA, and treated with TGFβ2 (5 ng/ml) for 48 h. (**b**) Real-time PCR analysis of Snail, Slug, and ZEB1 mRNA levels in LECs transfected as indicated in ***a***. (**c**) Real-time PCR analysis of FN, Col IV, vimentin, and N-cadherin mRNA levels in LECs exposure to TGFβ2 with or without different concentrations of DAPT (1.25, 2.5, 5, and 10 μM) for 48 h. (**d**) Real-time PCR analysis of Snail, Slug, and ZEB1 mRNA levels in LECs exposure to TGFβ2 with or without DAPT (2.5 μM) for 48 h. * *P*<0.05; ** *P*<0.01.

**Figure S7 Blockade of Notch pathway with DAPT suppresses LECs migration.** (**a**) Wound healing analysis of LECs migration after treated with or without DAPT (2.5 μM) for 48 h. Scale bar: 100 μm. (**b**) Quantification of the area of the remaining wound per field (n=18 randomized fields per group). * *P*<0.05. (**c**) EdU staining analysis of LECs proliferation after treated with or without DAPT (5.0 μM) for 48 h. Scale bar: 40 μm. (**d**) Quantification of EdU positive cells (n=24 randomized fields per group). *NS*: not significant.

**Figure S8 Jagged-1/Notch signaling pathway is activated in injury-induced ASC model in vivo.** The anterior capsules of mouse lenses were punctured with a needle and 1μl of 80 μM of DAPT were injected into the anterior chambers of the eyes immediately after injury. One and three days later, lenses were harvested for real-time PCR analysis of Jagged-1, Jagged-2, Notch-1, Notch-2, Notch-3, Hes-1, and Hey-1 mRNA levels. # *P*<0.05; ## *P*<0.01, *verus* the control group. * *P*<0.05; ** *P*<0.01, *verus* the DMSO treatment group.

**Figure S1**

**Figure S2**


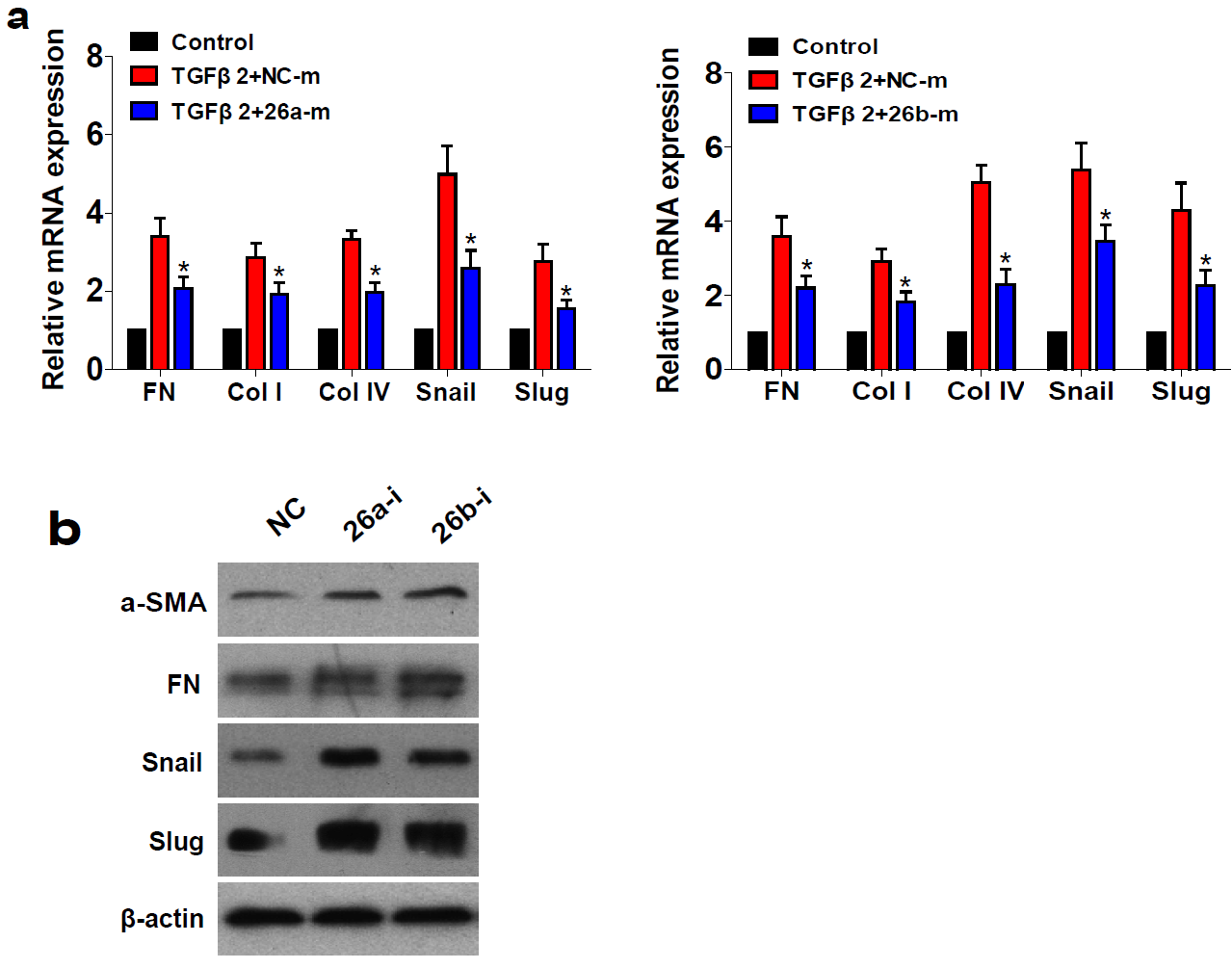


**Figure S3**

**Figure S4**

**Figure S5**

**Figure S6**

**Figure S7**

**Figure S8**
